# Mesenchymal Stromal Cells (MSCs): An Ally of B-Cell Acute Lymphoblastic Leukemia (B-ALL) Cells in Disease Maintenance and Progression within the Bone Marrow Hematopoietic Niche

**DOI:** 10.3390/cancers14143303

**Published:** 2022-07-06

**Authors:** Alessandra Fallati, Noemi Di Marzo, Giovanna D’Amico, Erica Dander

**Affiliations:** Centro Ricerca Tettamanti, Pediatric Department, University of Milano-Bicocca, Fondazione MBBM, 20900 Monza, Italy; a.fallati@campus.unimib.it (A.F.); n.dimarzo@campus.unimib.it (N.D.M.); e.dander@asst-monza.it (E.D.)

**Keywords:** B-cell acute lymphoblastic leukemia (B-ALL), mesenchymal stromal cells (MSCs), bone marrow (BM) niche, microenvironment targeting

## Abstract

**Simple Summary:**

B-cell acute lymphoblastic leukemia (B-ALL) is the most common pediatric cancer. Even though the cure rate actually exceeds 85%, the prognosis of relapsed/refractory patients is dismal. Recent literature data indicate that the bone marrow (BM) microenvironment could play a crucial role in the onset, maintenance and progression of the disease. In particular, mesenchymal stromal cells (MSCs), which are key components of the BM niche, actively crosstalk with leukemic cells providing crucial signals for their survival and resistance to therapy. We hereby review the main mechanisms exploited by MSCs to nurture and protect B-ALL cells that could become appealing targets for innovative microenvironment remodeling therapies to be coupled with classical leukemia-directed strategies.

**Abstract:**

Mesenchymal stromal cells (MSCs) are structural components of the bone marrow (BM) niche, where they functionally interact with hematopoietic stem cells and more differentiated progenitors, contributing to hematopoiesis regulation. A growing body of evidence is nowadays pointing to a further crucial contribution of MSCs to malignant hematopoiesis. In the context of B-cell acute lymphoblastic leukemia (B-ALL), MSCs can play a pivotal role in the definition of a leukemia-supportive microenvironment, impacting on disease pathogenesis at different steps including onset, maintenance and progression. B-ALL cells hijack the BM microenvironment, including MSCs residing in the BM niche, which in turn shelter leukemic cells and protect them from chemotherapeutic agents through different mechanisms. Evidence is now arising that altered MSCs can become precious allies to leukemic cells by providing nutrients, cytokines, pro-survivals signals and exchanging organelles, as hereafter reviewed. The study of the mechanisms exploited by MSCs to nurture and protect B-ALL blasts can be instrumental in finding new druggable candidates to target the leukemic BM microenvironment. Some of these microenvironment-targeting strategies are already in preclinical or clinical experimentation, and if coupled with leukemia-directed therapies, could represent a valuable option to improve the prognosis of relapsed/refractory patients, whose management represents an unmet medical need.

## 1. Introduction

### 1.1. Mesenchymal Stromal Cells (MSCs) as Key Regulators of the Healthy Hematopoietic Bone Marrow Niche

The bone marrow (BM) niche is a complex and highly specialized microenvironment consisting of different cell types, extracellular components, such as the extracellular matrix (ECM), chemical and physical factors. The BM microenvironment is not a unique niche, but a collection of several districts: to date, an endosteal and a perivascular niche have been described, where hematopoietic stem cell (HSC) functions are finely tuned due to the interaction with several cell subsets [1,2]. The BM hosts several subpopulations of hematopoietic and non-hematopoietic origin, including vascular cells, endothelial cells, megakaryocytes, macrophages and MSCs. All these cell subsets cooperate through the secretion of soluble signals, including cytokines, hormones, growth factors and through the expression of receptors and adhesion molecules to the regulation of hematopoiesis [3,4].

#### 1.1.1. MSCs: Main Characteristics

MSCs are versatile stem cell population due to their capacity to differentiate into bone (osteoblasts), fat cells (adipocytes) and cartilage (chondrocytes), with a key role in HSC maintenance and hematopoiesis regulation, BM niche composition and bone growth [5]. MSCs are characterized by the ability to grow in vitro as adherent cells, characterized by a fibroblast-like morphology. Phenotypically they are characterized by the high expression of CD73, CD90 and CD105 surface antigens, and by the absence of CD45, CD34, CD14, CD11b, CD79a, CD19 and human leukocyte antigens class II (HLA II) on their membrane [6]. Different studies have shown that MSCs have strong immunoregulatory properties both in vitro and in vivo [7,8]. MSCs are able to modulate innate and adaptive immune cells such as T-, B-, and NK-cells, monocytes and dendritic cells, in response to surrounding pro- or anti-inflammatory stimuli, thus shaping the BM immune infiltrate.

Importantly, MSCs and their progeny (osteoblasts/osteocytes, chondrocytes and adipocytes) are structural components of both endosteal and perivascular niches. Within these compartments, they functionally interact with HSCs and more differentiated hematopoietic progenitor cells, regulating their fate in terms of quiescence and activation of cell fate determination, proliferation, self-renewal and differentiation [9,10,11].

#### 1.1.2. Role of MSCs in the Endosteal and Perivascular BM Niches

As mentioned above, MSCs-derived osteoblasts dynamically balance bone formation within the endosteal niche [12]. They regulate HSC proliferation and erythroid differentiation by osteopontin (OPN) and erythropoietin (EPO) production, thus deserving the title of key regulators of HSC maintenance in this compartment [13,14]. In addition, osteoblasts produce different cytokines and growth factors that regulate HSC homing, quiescence and mobilization, such as CXC-chemokine ligand 12 (CXCL12) [15], granulocyte colony-stimulating factor (G-CSF) [16], stem-cell factor (SCF) [17], annexin 2 [18] (ANXA2), angiopoietin 1 (ANG1) [19] and thrombopoietin (TPO) [20]. Besides osteoblasts, their differentiated form, osteocytes, embedded in the bone matrix and extending long projections to connect with surrounding cells, further contribute to control HSCs through the secretion of G-CSF [21]. The vascular niche, instead, is considered a highly vascularized and proliferative district where leukocytes can traffic in and out of the BM. It is believed that an exchange between the endosteal and the vascular niches is continuous and that the HSC pool is in the balance between trafficking, proliferation and quiescence [22]. Around arterioles and sinusoids, CXCL12-abundant reticular (CAR) perivascular stromal cells along with endothelial cells, which are part of the vessel structure, are the main sources of CXCL12 and further contribute to HSC fate determination and mobilization [23]. A recent study by Baccin and colleagues, by means of combined single-cell and spatial transcriptomics, has provided new insight into CAR cell subset classification [3]. Indeed, they have identified two new CAR subsets characterized by a different niche localization: Osteo-CARs and Adipo-CARs. Osteo-CARs express high osterix (Sp7) and low Leptin receptor (Lepr) levels and preferentially localize to arteriolar or non-vascular niches. Adipo-CARs, instead, are characterized by high levels of Lepr and localize around sinusoids, which span the entire BM, including the sub-endosteal niches. Overall, these cell subsets contribute to the secretion of several cytokines and chemokines, including CXCL12 [3].

#### 1.1.3. Role of Adipocytes in the Healthy Hematopoietic BM Niche

Even if an “adipocytic” niche has never been so-far described, BM adipocytes (BMA) play a crucial role within the BM microenvironment. They are a heterogeneous cell subset with distinct metabolism, lipid composition, secretory properties and also functional responses, depending on the location within the BM. Among all their pleiotropic roles, the most important function is storage and release of lipids depending on the energetic status. When energy intake is greater than expenses, adipocytes store fatty acids (FA) as triglycerides under the control of insulin. In a state where energy is required, catabolic hormones such as glucocorticoids, adrenaline and glucagon spur hydrolysis of triglycerides into FA and glycerol [24]. BMA also contributes to hematopoiesis by positively regulating HSC regeneration under stress conditions. After radiotherapy or chemotherapy, when physiological HSC nests are temporarily destroyed, MSCs rapidly initiate adipogenic differentiation and newly generated adipocytes create a temporary HSC niche to maintain the body’s basic hematopoietic function and promote HSC regeneration by secreting SCF [25].

The well-established role of MSCs and their progeny in regulating hematopoiesis and HSC maintenance has been recently extended to pathological contexts of altered hematopoiesis. A growing body of evidence suggests that the BM microenvironment, and MSCs in particular, can be as already mentioned key modulators of leukemic cell survival, maintenance and progression also in the case of leukemias, including B-cell acute lymphoblastic leukemia (B-ALL).

### 1.2. Acute Lymphoblastic Leukemia (ALL): A Genetic Disease with a Crucial BM Niche Contribution

Acute lymphoblastic leukemia (ALL) is the most common pediatric hematological disorder arising from either B-cell (B-ALL) or T-cell (T-ALL) progenitors. B-ALL is the most frequent type of childhood ALL with an annual incidence of 3–4 cases per 100,000 and peak incidence in children aged 2 to 5 years [26]. While 80% of ALL occurs in children, it represents a devastating disease when it occurs in adults [27]. The disease is characterized by an uncontrolled proliferation of lymphoid progenitors in the BM, disrupting normal hematopoietic functions and generating a large number of immature lymphocytes. The pathogenesis stands on solid genetic bases: in the last years, genomic studies proved a close interplay between inherited and somatic genetic alterations in the biology of the disease. Indeed, the combination of genetic susceptibility factors and exposure to environmental onco-promoters contribute to the manifestation of the disease [28]. Many genetic alterations have important implications for diagnosis and risk-stratification of ALL and for the use and development of novel and targeted approaches.

Indeed, the presence of chromosomal alterations can be linked to a favorable or unfavorable prognosis. One of the most common abnormalities is the t(12;21) chromosomal translocation, that occurs in 25% of pediatric B-ALL patients, and leads to the formation of the chimeric fusion gene *ETV6-RUNX1* and has been associated with a favorable disease outcome [29,30]. Among the genetic alterations associated with an adverse prognosis, *BCR-ABL1* (Philadelphia chromosome-positive, Ph+ ALL), *KMT2A* rearrangements, *TCF/HLF*, *IKZF1* plus are the most frequent [31,32]. More recently, a new subtype of B-ALL patients has been identified, namely Philadelphia chromosome-like (Ph-like) or BCR-ABL1-like patients, characterized by a gene expression signature similar to that of Ph+ ALL patients, but lacking the BCR-ABL1 oncoprotein [33]. In the adult B-ALL population, it has been shown that a prevalence of t(9;22) can range from 15–50%, increasing with age [34]. Ph-positivity has been historically linked to a very poor prognosis, with a 5-years survival inferior to 20% [35].

Risk-adapted multi-agent treatment strategies, also including drugs targeting some fusion gene products (e.g., Tyrosine Kinase Inhibitors, TKIs), allowed the overall survival in childhood B-ALL to increase from under 10% in the 1960s to about 83–93% in adolescents and in young children, respectively, as published in 2019 [28,36,37]. The advent of TKIs also marked a turning point also in the treatment of adult Ph + ALL patients, dramatically increasing the overall survival (OS) [38,39]. Despite this strong progress in disease treatment, the prognosis for refractory and relapsed (r/r) ALL patients remains poor [40]. Even if B-ALL is considered a genetic disease, nowadays, a growing body of evidence suggests that the BM microenvironment can provide an essential contribution to the maintenance, the response to treatment, the progression and possibly the development of the disease, independently from the presence of specific genetic lesions in hematopoietic cells. Recent literature consistently reinforces the idea that B-ALL cells can modify the BM microenvironment, creating a malignant niche able, in turn, to support their survival to chemotherapy, thus possibly giving rise to disease relapse. Leukemic cells crucially interact with several components of the BM microenvironment, including MSCs, which are key constituents of the BM hematopoietic niche [41]. The interaction between leukemic cells and MSCs is a true bidirectional crosstalk, which takes advantage of signals coming from the interaction of adhesion molecules, secretion of cytokines and chemokines, exchange of metabolites and subcellular components, such as mitochondria as discussed in detail along this review. It is well recognized that MSCs contribute to modulate the proliferation, survival, chemoresistance and invasive properties of leukemic cells. However, whether MSCs could promote the onset of B-ALL is still an open field of research. Concerning this point, some reports suggest that MSCs could also play a role in the pathogenesis of hematological malignancies. In this context, results coming from myelodysplastic syndrome (MDS) indicate that patient-derived MSCs, characterized by a disturbed differentiation program and a deregulated secretome, are essentials for the propagation of MDS-initiating stem cells in orthotopic xenograft models. This suggests a permissive role of MSCs in the malignant evolution of MDS [42]. Moreover, some reports indicate that specific MSC alterations can influence the development of myeloid neoplasms in mice [43,44]. In 2007, Walkley and colleagues demonstrated that the transplantation of a BM microenvironment deficient for retinoic acid receptor γ (RARγ) was sufficient to initiate myeloproliferative syndromes in wild type mice. This reveals the capability of the microenvironment to be the sole cause of these hematopoietic disorders [45]. Alongside, Kode et al. proved, for the first time, that a single genetic defect, namely a β-catenin activating mutation in osteoblasts, is sufficient to promote the onset of acute myeloid leukemia in mice [46]. A causative function played by MSCs in the pathogenesis of B-ALL still needs to be proven. However, it is clear that these cells are essential contributors in the definition not only of the healthy hematopoietic BM niche, but also of a malignant niche, creating a “fertile” environment for the survival, expansion and promotion of leukemic cells, as hereafter reviewed.

## 2. MSCs as Key Modulators of Leukemia Onset, Maintenance, Progression and Drug Resistance within the B-ALL BM Niche

**Figure 1:**
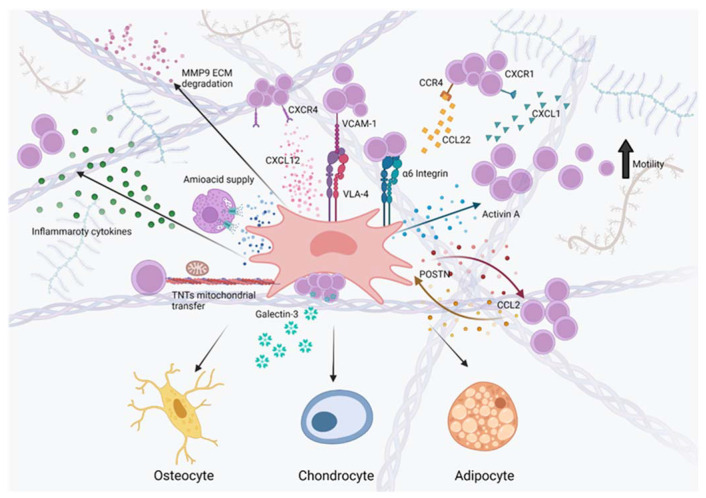
Role of mesenchymal stromal cells (MSCs) within the leukemic bone marrow (BM) niche. MSCs and their progeny including osteocyte/osteoblasts, adipocyte and chondrocyte (bottom) are key elements of B-cell acute lymphoblastic leukemia (B-ALL) BM niche. They interact through different mechanisms with leukemic cells protecting them from chemotherapy and providing signals that promote their survival and proliferation. Within the leukemic niche, MSCs secrete high levels of Activin A potentially promoting the migration of B-ALL cells to extramedullary sites (right, light blue dots). In turn, the high levels of Activin A downmodulate the production of CXCL12, possibly causing the displacement of healthy hematopoiesis in favor of leukemic cells. Other chemokine axes important for leukemia progression are represented by CXCR1/CXCL1 (blue triangles) and CCR4/CCL22 axis (yellow squares), which contribute to the recruitment of B-ALL cells into the BM niche. MSCs also contribute to the definition of the inflammatory environment, found in the leukemic BM niche, by secreting pro-inflammatory cytokines (left, green dots) including interleukin (IL)1β, tumor necrosis factor (TNF)α and IL6. The inflammatory environment increases the production of matrix metalloproteinases, such as MMP9 (violet dots), which may promote extracellular matrix (ECM) degradation and leukemia dissemination. MSCs also play a key role in protecting leukemic cells from drug-induced amino acid shortage and oxidative stress by providing nutrients such as asparagine (blue dots) and by transferring mitochondria and organelles via tunneling nanotubes (TNTs, left side). MSCs can also promote chemoresistance through cell–cell contact through very late antigen-4 (VLA-4), integrin α6 and Galectin (Gal)3-dependent pathways. The leukemic BM is characterized by high levels of MSCs-derived periostin (POSTN, right, red dots), which increase the expression of CCL2 (right, yellow dots) by leukemic cells and promote leukemia proliferation and adhesion to MSCs. Consequently, B-ALL-derived CCL2 induces the expression of POSTN, creating a self-reinforcing loop. Created with BioRender.com (accessed on 13 May 2022).

Literature data indicate that leukemic cells are able to hijack the normal BM microenvironment to create malignant niches, where they acquire a selective advantage over their normal counterpart and are protected from treatment. Recent studies have revealed the importance of MSCs in mediating these phenomena. Indeed, altered MSCs can exploit different mechanisms to shelter leukemic cells and protect them from chemotherapeutic agents. Evidence is now arising that MSCs can provide nutrients, cytokines, pro-survival signals and exchange organelles to sustain and promote leukemia progression. The pro-leukemic role of MSCs can also be achieved indirectly by shaping the BM niche immune infiltrate. Moreover, MSC can take an active part in the process of leukemogenesis: in case of some genetic lesions, such as the t(12;21), increasing evidence points to a contribution of MSC to the creation of a BM microenvironment that can favor the transition from a pre-leukemic state to overt disease manifestation. Hereafter, we depict the main mechanisms used by MSCs to support pre-leukemic clones and nurture and protect B-ALL cells (Figure 1). 

### 2.1. MSCs Contribute to B-ALL Pathogenesis and Progression by Creating a Leukemia-Supportive BM Microenvironment Rich of Transforming Growth Factor β (TGF β) Family Molecules and Inflammatory Mediators

MSCs are very plastic cells able to adapt their secretome based on external stimuli. Recent literature suggests that under inflammatory conditions (e.g., during infections), MSCs can contribute to the formation of a leukemia permissive microenvironment by the release of altered levels of transforming growth factor β (TGF β) family members. In detail, deregulation of these soluble factors can promote the transition from a pre-leukemic to a leukemic state and the progression of the disease itself as described below. Moreover, MSCs, in concert with other immune cells, participate to the formation of a BM microenvironment rich in pro-inflammatory factors, which is a typical feature of the B-ALL BM niche.

#### 2.1.1. MSCs Contribute to the Alteration of TGFβ Family Members, Implicated in B-ALL Onset and Progression

The transforming growth factor β (TGFβ) is an important regulator of hematopoiesis and plays a fundamental role in regulating the balance between proliferation and differentiation in hematopoietic cells. Alterations in the TGFβ pathway are often found in leukemia, though disruption of TGFβ is not sufficient to initiate malignant transformation. However, the loss of TGFβ signaling either via mutational inactivation of components of the signaling pathway, or by downmodulation of their expression may be a critical second hit that contributes to disease manifestation [47]. 

In the case of t(12;21), the chromosome translocation generates a preleukemic clone, which is insufficient to give rise to full blown leukemia. Indeed, additional genetic alterations are necessary for the transition of silent pre-leukemic cells to overt leukemia. In detail, the expression of the fusion gene *TEL-AML1* in the pro-B cell line BaF3 causes a slowing of cell growth compared to control and confers resistance to the growth-inhibitory effect of TGFβ by blockade of SMAD signaling. By this mechanism, *TEL-AML1*–positive BaF3 cells may acquire a selective advantage over their normal counterparts in conditions of immune or inflammatory response where TGFβ is engaged to dampen the B and T cell expansion [48]. 

Recently, our group has demonstrated a similar role for another member of the TGFβ family, that is Activin A. Indeed, we have demonstrated that Activin A, whose production by MSCs is induced under inflammatory conditions, is able to inhibit the proliferation of normal progenitor B cells, but not that of preleukemic *TEL-AML1*−positive clones, thereby providing them with a selective advantage [49]. 

In addition, we have found Activin A to be strongly overexpressed in the BM plasma of pediatric B-ALL patients at diagnosis compared to healthy donors (HD), and that ALL-MSCs produce higher levels of Activin A, compared to HD-MSCs. Importantly, the expression of this molecule by MSCs is strongly increased upon co-culture with primary B-ALL cells, indicating that its secretion is specifically driven by leukemia. Another noteworthy aspect is that MSCs-secreted Activin A confers a migratory advantage to B-ALL, both in vitro and in a xenograft mouse model. These data are in line with the well-recognized role of this molecule in invasiveness and metastasis in the context of several solid tumors [50,51,52]. In detail, Activin A is able to enhance the migration of primary leukemic cells in response to low levels of the chemoattractant CXCL12, while inhibiting the migration of healthy CD34+ cells [53]. In this way, in the BM of B-ALL patients, the low levels of CXCL12 could favor the expansion and retention of leukemic cells to the detriment of their healthy counterparts. Thus, the high levels of Activin A found in the BM of B-ALL patients may on one side promote the dissemination of leukemic cells to extramedullary sites, and on the other side, promote the displacement of HSCs from protective BM niches [53].

Another TGFβ family member deregulated within the B-ALL BM niche is the bone morphogenic protein 4 (BMP4). In this case, ALL-MSCs also secrete higher quantities of BMP4, compared to their healthy counterpart and compared to MSCs isolated from out of therapy patients. Moreover, BMP4 production is further induced upon co-culture with the leukemic cell line REH [54]. These findings make TGFβ family members interesting subjects of study to increase the current knowledge on leukemia pathogenesis and identify new therapeutic targets. Indeed, neutralizing antibodies to TGFβ, inhibitors to TGFβ Receptor I (TβRI), antisense nucleotides and ligand traps (for TGFβ and other family members) are currently in clinical trial for the treatment of human cancers making them easily transferable to the context of B-ALL. For example, TβRI inhibitor Vactosertib is currently in clinical trials (NCT05400122, Table 1) for the treatment of colorectal cancer patients and patients with r/r hematologic malignancies.

#### 2.1.2. MSCs Contribute to the Creation of a Highly Pro-Inflammatory and Pro-Leukemic Cytokine Milieu within the B-ALL BM Niche

The contribution of inflammation to cancer development has been extensively addressed in the literature. Chronic, persistent and unresolved inflammation has been associated with an increased risk of malignancies in several types of solid cancers [56]. In the context of B-ALL, how the inflammatory environment contributes to the transition of pre-leukemic clones to overt leukemia in presence of the TEL-AML1 rearrangement has been described. Indeed, mice bearing the t(12;21) developed B-ALL when exposed to common pathogens [57]. On the same line, TEL-AML1 cells repeatedly stimulated with lipopolysaccharide (LPS), gave rise to full blown leukemia when transplanted in mice [58]. Besides leukemogenesis, an inflammatory environment is believed to also contribute to disease maintenance and progression. Indeed, the BM of ALL patients is particularly rich in pro-inflammatory cytokines at diagnosis as demonstrated by the high levels of Interleukin (IL)6, IL1β and Tumor Necrosis Factor (TNFα) that have been detected in the BM of B-ALL patients at diagnosis [53]. Importantly, MSCs contribute to the definition and maintenance of this inflammatory state by increasing the secretion of several cytokines such as IL1α, IL6, IL12p70 and TNFα, as well as interferon type I and type II [59]. Furthermore, it has been demonstrated that stimulation of MSCs and endothelial cells with a cocktail of IL6, IL1β and TNFα is able to induce the production and secretion of CCL2 and CX3CL1 [60], which could contribute to the recruitment of monocytes in the BM and to the M2-like polarization of macrophages as observed in patients’ biopsies [60]. Recently, our group has also demonstrated for the first time an increase in the complement fraction C5a, a macrophage chemoattractant and M2-polarizing factor, in the BM plasma of B-ALL patients. This increase is accompanied by a reduced expression of the long pentraxin 3 (PTX3), which is a well-known regulator of inflammatory response. PTX3 is implicated in the regulation and activation of the complement cascade and its deficiency has been associated with increased cancer-related inflammation in several solid tumors [61]. Interestingly, in B-ALL patients, we have observed an inverse correlation between PTX3 levels and leukemic infiltrate in the BM, which may lead to complement activation with subsequent release of C5a. Moreover, even if a direct link between the pro-inflammatory cytokine milieu characteristic of B-ALL and cellular senescence has never been proven, it is well known that senescent cells release pro-inflammatory cytokines, chemokines and proteases, generally known as Senescence-Associated Secretory Phenotype (SASP). Moreover, SASP contributes to tumor progression by creating an immunosuppressive environment [62]. In this regard, a few reports suggest the activation of a senescence program in healthy MSC co-cultured with B-ALL cell lines, characterized by morphology alterations, increased senescence-associated-galactosidase (SA-GAL) activity, upregulation of p53 and p21, cell-cycle arrest and reduced clonogenicity [63,64]. This process is thought to be strictly dependent on MSC stimulation by leukemic cells and reversible. Interestingly, MSC isolated from the BM of B-ALL patients revealed the above-mentioned senescence-related features [63]. 

Overall, these findings suggest that the contribution of inflammation to the development of cancer is not restricted only to solid tumors, but it may also play a pivotal role in the context of hematological malignancies. Indeed, the stromal compartment may have a central role in the regulation of the inflammatory mediators.

### 2.2. MSCs/Leukemia Crosstalk Is Essential for the Creation of Altered Pro-Leukemic Chemokine Axes within the B-ALL BM Niche

Cytokines and chemokines secreted from different components of the BM play an essential role in regulating the homeostasis of the hematopoietic microenvironment and the fate of HSCs by providing the stimulus for proliferation, survival, self-renewal, differentiation and functional activation [65]. Leukemic cells are able to hijack the BM microenvironment and alter these chemokine axes to create a malignant niche, which better support neoplastic cell survival and proliferation at the detriment of healthy HSCs [66]. Indeed, several immune and non-immune cell subsets, such as macrophages, dendritic cells and vascular cells orchestrate the levels of BM chemokines. Focusing on the stromal contribution, it has been demonstrated that MSCs-derived chemokines are able to influence the migration of leukemic cells, enhance their metastatic potential, or recruit immune cells to create a leukemia-promoting microenvironment. Chemokine pattern alterations are characteristic of all forms of leukemia, including B-ALL and their study may be instrumental in identifying novel prognostic markers and druggable targets to improve leukemic cell killing.

#### 2.2.1. CXCL12/CXCR4 Axis

Among the different chemokine axes regulating the BM niche, the CXCL12/CXCR4 axis has been demonstrated to play a relevant role in the development and progression of B-ALL [67]. As previously mentioned, the CXCL12 chemokine, produced by MSCs, has a key role in the homing and maintenance of the HSC pool in the BM niche [23] and in promoting their survival and self-renewal [68]. Indeed, it has been demonstrated that disturbance of the CXCR4/CXCL12 axis by treatment with CXCR4 antagonists promotes the mobilization of HSCs to the periphery [69] and prevents their engraftment in the BM of NOD/SCID mice [70]. In the same way, leukemic cells share this mechanism for homing to CXCL12-secreting niches within the BM. Interestingly, in B-ALL, CXCL12 levels in the BM of patients at diagnosis are significantly lower compared to healthy controls or patients in the remission phase [53,67]. Moreover, leukemic cells have an increased expression of CXCR4-receptor, and its levels are correlated with an unfavorable clinical outcome and increased risk of relapse. An elegant study of Colmone and colleagues has demonstrated that leukemic proliferation occurs preferentially within CD34+ cell homing niches and alters the stromal microenvironment creating a malignant niche of heavy tumor growth characterized by particularly low levels of CXCL12, that cause the displacement of normal HSCs [71]. Accordingly, the high levels of Activin A found in the BM of leukemia patients are able to enhance the migration of B-ALL cells toward low doses of CXCL12, while hindering the migration of healthy CD34+ cells [53].

Interestingly, the CXCL12/CXCR4 axis also drives the colonization of extramedullary tissues where leukemic cells find sanctuary sites that protect them from therapy. Indeed, Crazzolara and colleagues reported a correlation between high CXCR4 expression on leukemic cells and extramedullary organ infiltration [72], whilst Kato and colleagues, using a xenograft mouse model, have demonstrated that liver dissemination of leukemia is driven by the CXCL12/CXCR4 axis [73].

Therefore, the CXCL12/CXCR4 axis contributes on one side to the retention of malignant cells in the BM, on the other it favors their dissemination to distant organs, where they find sanctuary sites such as testis and central nervous system, (CNS) where they are protected from systemic therapy and may give rise to relapses.

Given the importance of CXCL12/CXCR4 signaling in B-ALL progression, the pharmacological inhibition of this signaling has been proposed to disrupt the cross talk between B-ALL cells and the protective stroma. Plerixafor (AMD3100) and BKT (Table 1) are small molecule drugs acting as CXCR4 antagonists that were shown to be able to overcome the stroma mediated protection from chemotherapy. Indeed, addition of Plerixafor or BKT140 to NALM6-BM-MSC co-culture sensitized NALM6 cells to either Vincristine or Dexamethasone [74]. Moreover, prolonged administration of AMD3100 to NOD/SCID mice engrafted with B-ALL cells significantly reduced the number of leukemic cells in peripheral blood (PB), spleen, liver and kidney, demonstrating that disruption of CXCL12 signaling inhibits the in vivo growth and dissemination of leukemic cells [75]. In line with these results, CXCR4 genetic deletion in NALM6 cells reduced the leukemic infiltration in BM and the numbers of circulating leukemic cells, besides prolonging the survival of the mice [74]. Despite the promising pre-clinical results obtained using CXCR4 antagonists, the application of these drugs in clinic did not bring the expected results. Indeed, the phase I/II clinical trial (NCT01319864) with CXCR4 antagonist as a chemosensitizing agent in patients with r/r Acute Myeloid Leukemia or r/rALL did not produce obvious benefit over chemotherapy alone [76,77]. This suggests that the CXCR4/CXCL12 axis is not the only one exploited by leukemic cells to hijack BM marrow niches.

#### 2.2.2. Other Chemokine Axes Involved in B-ALL Pathogenesis

Indeed, De Rooji and colleagues, have demonstrated that B-ALL cells are able to create a self-reinforcing malignant niche independently from CXCL12. They observed that CXCR4 inhibition by AMD3100 did not affect the migration of B-ALL cells toward ALL-MSC co-culture, suggesting that B-ALL cells create a leukemic niche that attract leukemic cells in a CXCR4/CXCL12 independent manner. Accordingly, they identified other chemokine axes which could contribute to the recruitment of B-ALL cells and inhibition of CD34+ cells migration in BM-niche, such as CCR4 and CXCR1/2 axes. Indeed, they demonstrated that co-culture of MSCs with B-ALL cells upregulates the expression of CCR4 and CXCR1/2 ligands, namely CCL22 and CXCL1 by MSCs. Accordingly, high levels of CXCL1 were found in the BM of B-ALL patients at diagnosis [78].

The deregulation of several MSCs-derived cytokines upon leukemia engraftment has also been underlined by De Vasconcellos and colleagues, who observed that primary ALL cells are able to alter the secretome of BM-MSCs increasing the release of a wide array of cytokines, including CXCL10, CXCL8, CCL2, CXCL11, CCL7 and CXCL2. In line with these results, they found significantly higher levels of CXCL8 and CCL2 in the BM of B-ALL patients at diagnosis compared to normal BM samples [79]. Concerning the possible impact on disease, they observed that CCL2 and CXCL8 were able to increase the adhesion of ALL cells to BM stromal cells and promote survival and proliferation of the latter, suggesting that the leukemic cells modulate the function of tumor-supportive BM-MSCs. Importantly, a study by Kerr and colleagues correlating BM chemokines levels with disease outcome identified CCL2 as a late biomarker of poor prognosis [80].

All these results suggest that the protective microenvironment found in the BM niche may be the result of the alteration of multiple signaling pathways and interfering with these axes might be an alternative or additional way to disrupt the B-ALL niche.

### 2.3. MSCs Exchange Amino Acids, Metabolites and Mitochondria with B-ALL Cells to Promote Chemoresistance and Provide an Antioxidant Defense

In B-ALL, leukemic cells proliferate in an uncontrolled manner and accumulate in the BM and, through the peripheral circulation, into extramedullary target organs such the spleen, the liver, the lymph nodes and the CNS. In general, rapidly dividing cells have an increased demand in nutrients for the synthesis of proteins, nucleic acids, and lipids to sustain their growth and proliferation. In some cases, upon metabolic reprogramming, cancer cells become dependent on extracellular nutrients to survive [81]. In the context of hematologic malignancies, B-ALL cells develop dependency on specific amino acids for their survival, such as asparagine and arginine, therefore interfering with amino acid availability can be detrimental for tumor cells. In the last few years, it has been shown that MSCs can support leukemia cell growth and mediate chemoprotection by providing amino acids, metabolites and even entire organelles to B-ALL cells. This is based upon a bi-directional exchange of soluble factors, extracellular vesicles (EVs) and nanotube-based connections.

#### 2.3.1. MSCs Protect B-ALL Cells from Chemotherapy-Based Amino Acid Depletion by Providing Ready-to-Use Amino Acids

Resistance of leukemia cells to chemotherapy is a major clinical challenge in the treatment of B-ALL. Recent findings indicate that MSCs metabolic plasticity plays a pivotal role in drug resistance, by regulating cancer cell metabolism and the metabolic environment. In this context, asparaginase (ASNase) is part of polychemotherapy regimens used for B-ALL treatment [82,83] and in advanced phase clinical trials for other hematological malignancies, such as actute myeloid leukemia (AML) and T-ALL (Table 1). Indeed, leukemic cells are auxotrophic for asparagine, which means that they strictly depend upon extracellular asparagine to survive, due to the low expression of asparagine synthetase (ASNS) [84]. Therefore, the anti-leukemic effect of ASNase depends on its ability to rapidly exhaust the pool of circulating asparagine, leading to a subsequent depletion of intracellular Asn in leukemic cells, thus producing nutritional stress, proliferative arrest and, eventually, death of leukemic cells [85]. Of note, expression levels of ASNS in leukemic cells are not correlated with a clinically poor response to the drug [86], and the molecular basis of asparaginase resistance remains poorly understood. A growing body of evidence is now emerging about the protective role of MSCs against ASNase cytotoxicity. Indeed, when co-cultured with MSCs, ALL blasts are effectively protected from ASNase-induced cytotoxicity. This effect can be achieved through different mechanisms. Iwamoto and colleagues demonstrated that ASNS silencing in MSCs, by RNA interference, severely hindered their capability to protect ALL cells from ASNase toxicity, suggesting that the protection could be mediated by asparagine biosynthesis and its secretion in the BM microenvironment [87]. Interestingly, as recently demonstrated by Chiu and colleagues, patient-derived MSCs (ALL-MSCs) are more efficient in protecting leukemic cells from ASNase treatment compared to healthy donor-derived MSCs [88]. This increased ability has been ascribed to a significant overexpression of the transporter SNAT5 in ALL-MSCs, which, by mediating Asn and Gln transport, could contribute to maintain Asn availability in the BM microenvironment, despite ASNase treatment. Notably, B-ALL blasts play an active role in this amino acid trade-off. Indeed, upon Asn shortage, leukemic cells upregulate glutamine synthetase increasing the production of Gln, which is then secreted in the extracellular space and uptaken by MSCs to synthetize Asn. This metabolic crosstalk between leukemic cells and BM-MSC could represent a key mechanism within the leukemic BM niche, exploited by leukemic blasts to resist ASNase-dependent chemotherapy [88].

Recent findings suggest that leukemic cells may be dependent also on extracellular arginine for survival. Pre-clinical studies have demonstrated that solid tumors (melanoma, hepatocellular carcinoma and prostate adenocarcinoma) are vulnerable to therapeutic arginine depletion. Indeed, in phase I/II clinical trials, administration of PEGylated (PEG) recombinant human arginase (BCT-100, Table 1) has shown efficacy in patients with advanced hepatocellular carcinoma [89]. Recent in vitro and in vivo studies have highlighted the importance of arginine depletion also in the context of B-ALL. BCT-100-based arginine depletion reduced disease burden and prolonged survival in ALL murine xenografts [90] and is currently under clinical trial for r/r ALL (NCT03455140). Although in B-ALL there are no studies concerning MSCs-mediated protection from arginine depletion, in T-ALL it has been demonstrated that the efficacy of BCT-100 is significantly reduced in presence of MSCs, which provide to leukemic cells soluble factors involved in L-arginine metabolism [90]. These findings could open the way to new studies on the role of MSCs in protecting B-ALL from arginine depletion.

#### 2.3.2. MSCs Protect B-ALL Cells from Chemotherapy by Means of a Tunneling Nanotubes-Based Transfer of Mitochondria and Other Cellular Components

Besides providing amino acids, MSCs can protect B-ALL cells from chemotherapeutic agents also by exchanging various types of cargos along tunneling nanotubes (TNTs). TNTs are membrane protrusions with a skeleton of F-actin, that connect several neighboring cells at once [91]. They represent a novel kind of cell-to-cell communication used by different types of cells to exchange cytoplasmic molecules such as inositol trisphosphate [92], proteins or miRNA [93], but also organelles [94], pathogens [95] and cell-surface molecules. Communication through TNTs has also been described in hematological malignancies between leukemic cells and MSCs in the context of AML [96,97] and T-ALL [98]. Recently, TNTs have been recognized as a highly effective communication mechanism also in B-ALL, where they are able to mediate a bidirectional transfer of organelles and membrane components between MSCs and B-ALL cells [99]. Interestingly, disruption of TNTs by shaking or transwell inserts heavily impacts the survival of primary B-ALL blasts in presence of prednisolone [99], demonstrating the importance of TNTs in sustaining and protecting leukemic cells from chemotherapy. Recent findings have also demonstrated a transfer of endosome, mitochondria and autophagosome from B-ALL cells and MSCs through TNTs [100]. Most interestingly, the transfer of mitochondria along TNTs is able to protect leukemic cells from ROS-inducing agents. Indeed, depletion of mitochondria from MSCs severely impaired the rescue of SEM cell line from the chemotherapeutic agent, AraC. The importance of TNTs in mediating drug resistance has been demonstrated also in vivo in a xenograft mouse model of leukemia. Indeed, upon treatment with AraC, leukemic cells show an increase in mitochondrial mass which is partially prevented by administration of Vincristine, a microtubule inhibitor [101]. Vincristine is part of polychemotherapy regimens for the treatment of several pathologies including, among the others, acute leukemia, malignant lymphoma and non-Hodgkin’s lymphoma. In the case of B-ALL, its anti-leukemia action may be ascribed both to a mitotic arrest of proliferating leukemic cells and also to the disruption of the interaction with the supporting microenvironment.

#### 2.3.3. B-ALL Cells Secrete Large Amounts of EVs to Reprogram MSCs and Modify the Behavior of Surrounding Cells

Recent evidence demonstrates that B-ALL cells release a variety of EVs, different in terms of size and cargo, within the BM microenvironment that are internalized by stromal cells, both in vitro and in vivo. Literature data indicate that ALL-derived EVs induce a metabolic switch from oxidative phosphorylation to aerobic glycolysis in recipient stromal cells. These reprogrammed cells secrete lactate in the extracellular space, which is used by tumor cells as a source of energy, thus potentially facilitating leukemic cell survival [102,103]. The mechanisms underlying this metabolic reprogramming are still obscure. However, what is clear is that B-ALL EVs can contain a cargo that is rich in mRNA, miRNA, proteins and even cellular organelles such as mitochondria, lysosomes and Golgi [102]. However, MSCs are not the only target of B-ALL-derived EVs. In this regard, Georgievski and colleagues demonstrated that ALL-derived EVs target endogenous murine Hematopoietic Stem and Progenitor Cells (HSPCs) within BM, disturbing their quiescence and maintenance. In detail, metabolomics studies revealed that ALL EVs were particularly rich in cholesterol, which accelerated the mitochondrial activation and promoted the loss of quiescence in targeted HSPC. This resulted in the exhaustion of quiescent healthy HSPC, confirmed by a reduced ex vivo ability of forming colonies [104].

Importantly, leukemic cells themselves are a direct target of ALL-derived EVs. Indeed, it has been recently demonstrated that exosomes secreted by proliferating pre-B ALL cells can directly stimulate the growth of slow-growing clones [105]. In this regard, MSC-derived exosomes have been demonstrated to exert a pro-tumoral action in the context of several hematological malignancies, including multiple myeloma (MM), chronic lymphocytic leukemia and acute myeloid leukemia. In these pathologies, exosome protein and miRNA cargo proved to enhance proliferation, migration and drug-resistance of leukemic cells [106,107,108,109,110]. In the context of B-ALL, scientific evidence is still lacking; hopefully future studies will fill this gap.

### 2.4. MSC/Leukemic Cell Adhesion Activates Molecular Pathways Promoting B-ALL Maintenance, Chemoprotection and Progression

Leukemic cells, by responding to chemotactic stimuli within the BM niche, are able to gain contact with MSCs. Cell-to-cell contact between BM-MSCs and ALL cells depends on different types of adhesion molecules (e.g., CD44,intercellular adhesion molecule (ICAM), vascular cell adhesion molecule (VCAM), lymphocyte function-associated antigen 1 (LFA-1), selectins, galectins and other integrins) and leads to the subsequent activation of downstream signaling, such as nuclear factor kB (NF-κB), focal adhesion kinase (FAK), mitogen-activated protein kinase (MAPK) and phosphoinositide 3-kinase (PI3K/Akt). Experimental evidence has demonstrated that some adhesion pathways profoundly impact on leukemic cell protection from spontaneous and chemotherapy-induced apoptosis and disease progression [41,111,112,113]. Moreover, the interaction of leukemic cells with MSCs by means of adhesion molecules is able to trigger the activation of pro-survival and proliferative pathways not only in leukemic cells, but also in stromal cells, thus indicating a true bidirectional crosstalk with molecular consequences on both cell types [112]. Interestingly, the pharmacological inhibition of these adhesion molecules and their downstream signaling pathways, such as extracellular signal-regulated kinase (Erk, p38), PI3K/Akt in stromal cells proved to be sufficient in many cases to overcome drug resistance in leukemic cells, as discussed below [112,113,114,115].

#### 2.4.1. MSCs Chemoprotect Adherent B-ALL Blasts through Integrin-Mediated Mechanisms

One well described leukemia/MSC adhesive interaction, impacting on leukemic cell resistance to chemotherapy, is based upon the binding of the α4β1 integrin very late antigen-4 (VLA-4) to (VCAM-1). Jacamo R. and colleagues demonstrated that VCAM-1/VLA4-mediated contact between leukemic cells and MSCs is able to promote the activation of NF-kB signaling in BM-MSCs, and the upregulation of NF-kB target genes, including cytokines and chemokines such as CXCL8, IL6 and CCL2 and membrane VLA-4 and VCAM-1 themselves [112]. Interestingly, NF-kB blockade by means of chemical agents or overexpression of a super repressor form of IkBα (IkBα-SR), was able to revert, in vitro, the stroma-mediated resistance of leukemic cells to chemotherapeutic agents [112]. Furthermore, in an ectopic model of humanized BM niche generated in immunodeficient mice, leukemic cells engrafted within the ectopic niche were more sensitive to chemotherapy treatment if NF-kB signaling was blocked by overexpression of IkBα-SR in niche-composing MSCs [112]. Accordingly, inhibition of PKC-β isoforms, which are key components of the NF-kB pathway activated by VCAM-1/VLA-4 interaction in BM-MSCs, resulted in a detachment of almost 50% of co-cultured B-ALL cells and a reduction of leukemic cell survival [113]. Furthermore, treatment of MSC co-cultured B-ALL cells by means of a chimeric peptide (HKPS) (Table 1) directed against classical protein kinase (PK)C isoforms, namely HKPS, was able to increase leukemic cell chemosensitivity to dexamethasone, methotrexate and vincristine [113]. Interestingly, one possible mechanism is the inhibitory effect of HKPS on VLA-5 and ICAM-1 expression, usually upregulated in MSCs by B-ALL co-culture [113]. In line with these data, alpha4 integrin (ITGA4) mRNA, which encodes for one of the VLA-4 chains, when overexpressed in B-ALL patients with minimal residual disease (MRD) has been associated with a poor prognosis [114]. Moreover, Hsieh and colleagues demonstrated that alpha4 integrin conditional deletion or blockade, using the monoclonal antibody, Natalizumab (Table 1), was sufficient to sensitize primary B-ALL cells to chemotherapy in vitro and in vivo [114]. Because of its clinical usage for the treatment of osteosarcoma, multiple myeloma, multiple sclerosis and Crohn’s disease (Table 1), this drug could be easily translated also to the B-ALL setting. Besides VLA-4, other integrins have been implicated in B-ALL adhesion to MSCs and chemoresistance. One of them, the alpha6 integrin, already known for its ability to guide blast migration to the CNS and implicated in the persistence of MRD, was recently associated with B-ALL drug resistance [115]. Its blocking by means of an alpha6-targeting antibody (P5G10, Table 1) was able to induce apoptosis in primary B-ALL cells in vitro and to sensitize them to tyrosine kinase inhibition. A recent paper from Kihira and colleagues [41] shows that leukemia cells, expressing high levels of proteins in the cadherins and integrins pathways, upon adhesion to MSC acquire chemoresistance to commonly used anti-leukemic agents and undergo immunophenotype changes typical of cancer stem-like cells.

#### 2.4.2. MSCs Chemoprotect B-ALL Blasts through the Exosomal Release of the Adhesion Protein Galectin-3

Another adhesive protein regulating leukemic cell chemosensitivity that deserves a special mention is Galectin-3 (Gal-3), a β-galactoside-binding protein endowed with pleiotropic biological functions including cell–cell and cell–matrix adhesion [116,117,118]. In tumoral contexts, Gal-3 can exert a pro-tumoral role by impacting on tumor angiogenesis, cancer–matrix interaction, malignant cell metastasis, and drug resistance [119,120,121]. In the context of ALL, Gal-3 was found significantly increased, at the protein level, in the BM plasma of ALL patients compared to HDs [122] and, at the mRNA level, in BM mononuclear cells isolated from r/r ALL patients compared to patients responding to therapy [122]. Interestingly, Gal-3 is significantly upregulated in B-ALL cells upon contact with stromal cells, which can release it into exosomes [123]. Once internalized by ALL cells, Gal-3 can stimulate the transcription of endogenous *LGALS3* (Gal-3) mRNA in a sort of auto-activation transcription process. Interestingly, Hu and colleagues demonstrated that high expression of Gal-3 in B-ALL cells is associated with resistance to cytotoxic drugs, possibly by activating the Wnt/β-catenin pathway, and the transcription of oncogenes such as CyclinD-1, Survivin and c-Myc [124].

Overall, these findings suggest that targeting B-ALL/MSC adhesive pathways and the molecular signaling cascade activated downstream could be a valuable option to be coupled with conventional chemotherapy to potentiate its effect and to achieve a durable disease remission.

### 2.5. MSC/Leukemic Cell Crosstalk Instructs MSCs to Secrete Altered Matricellular Proteins to Create a Leukemia-Favoring ECM

Aberrant expression of ECM proteins by MSCs results in an alteration of adhesive properties of B-ALL cells, leading to leukemia progression and chemotherapy escape. In the last years, ECM has attracted ever growing attention since alteration of its composition and structure have been described in the context of several solid cancers. Indeed, tumor ECM is more abundant, denser and stiffer, and strongly differs from the ECM of surrounding normal tissue also in terms of composition and post-translational modification. So far, as it concerns B-ALL, even if an in-depth analysis of the ECM composition has not been completed, some ECM components have been shown to be deregulated, as a result of the leukemia/MSCs crosstalk.

In this regard, it has been recently demonstrated that within the B-ALL niche, MSCs produce increased levels of the matricellular protein, periostin (POSTN), impacting on leukemia progression. Interestingly, Ma and colleagues demonstrated that B-ALL cells are responsible for stimulating BM-MSCs to produce POSTN, which was found to be abundant in the BM plasma of B-ALL patients at diagnosis [125]. POSTN, in turn, increases the expression of CCL2 by leukemic cells and promotes leukemia cell proliferation and adhesion to BM-MSCs through the integrin-ILK-NF-κB signaling pathway. Consequently, B-ALL-derived CCL2 induces the expression of POSTN creating a self-reinforcing loop [126]. It is worth noting that POSTN knockout in a leukemia xenograft mouse model severely impaired leukemia cell proliferation and dissemination, suggesting that the analysis of leukemic ECM and its alterations could reveal new clinically relevant pharmacological targets.

Another matrix glycoprotein deregulated within the leukemic BM niche is OPN. This glycoprotein, endowed with pro-tumoral functions, as demonstrated in the contest of several solid and hematological cancers [127,128,129,130,131], can act both as a soluble cytokine or chemokine and as an adhesion molecule within the BM ECM. In the case of B-ALL, Ma and colleagues [132] demonstrated, by means of a 3D organotypic device, recapitulating the complexity and heterogeneity of the BM niche, that leukemic cells can upregulate, upon coculture, OPN expression in MSCs and that OPN can, in turn, crucially mediate leukemic cell adhesion to MSCs. Based on the physiological ability of OPN to promote HSC dormancy within the endosteal niche [13,133,134], it has been hypothesized that OPN may play a similar role also in the context of B-ALL. Indeed, it has been demonstrated that even though OPN is not able to directly induce dormancy of leukemic cells, and it recruits and anchors B-ALL cells within prodormancy niches, where other factors can promote cell cycle arrest, protecting them from cytotoxic drugs that act on actively cycling cells [135]. Accordingly, pharmacological blockade of OPN proved to be an effective strategy to enhance the sensitivity of leukemic cells to Ara-C-based chemotherapy, as demonstrated in a B-ALL mouse model [135], suggesting a possible translation of this result to a clinical contest.

The importance of leukemia–stroma crosstalk in ECM remodeling and leukemia progression has been underlined also by Verma and colleagues [136] who explored the role of matrix metalloproteinase (MMP) in the leukemic BM and its contribution to disease progression. In the context of solid tumors, MMPs-mediated ECM degradation favors cancer cell migration into the ECM, thereby causing local invasion and metastasis [137,138]. Recently, Verma and colleagues have described a similar role for MMP9 also in B-ALL. Indeed, B-ALL cells are able to instruct the BM microenvironment to degrade ECM thus favoring leukemia progression [136]. In particular, they demonstrated that leukemic cells, through the secretion of TNFα, are able to induce the release of MMP9 by MSCs, thus promoting matrix degradation and favoring leukemia dissemination [136]. These data, also supported by several reports about the key role of MMPs in the progression of solid and hematological cancers [139,140,141], suggest that MMP9 could be targeted in combination with existing therapies to improve B-ALL management.

Recent findings have drawn the attention on the secreted protein acidic and rich in cysteine (SPARC), a matricellular protein endowed with anti-adhesive properties, as another crucial matrix component able to impact on the adhesion of leukemic blasts to MSCs and consequently on their chemoprotection. In this regard, it has been demonstrated that SPARC inhibition by means of small interfering RNA is able to promote the adhesion of NALM6 cells to BM-MSCs [142]. Moreover, BM-MSCs isolated from B-ALL patients showed lower SPARC expression than their healthy counterparts, suggesting that B-ALL cells could reduce SPARC expression in BM-MSCs to promote their survival through adhesion. Accordingly, Iwasa and colleagues demonstrated that the proteasome inhibitor Bortezomib (Bor) (Table 1) reduces the adhesion of B-ALL cells to MSCs by upregulating the expression of SPARC in BM-MSCs. Interestingly, administration of Bor is highly effective in diminishing leukemic cell proliferation and restoring their chemosensitivity in vivo [142]. Bor could be an interesting candidate as adjuvant therapy in B-ALL, since it is already amply used in the clinical setting (e.g., Phase III for myeloma patients [143]). Its main mechanism of action is thought to be the reversible inhibition of the 26S proteasome, leading to cell cycle arrest and apoptosis of cancer cells. However, multiple mechanisms, including the one mentioned above, may be involved in the anticancer activity of Bor.

These preliminary reports indicate the importance of investigating the contribution of B-ALL MSCs in the creation of a leukemia permissive ECM.

### 2.6. MSCs Shape the Immune Microenvironment of the B-ALL BM Niche

MSCs can affect the BM immune cell infiltrate by interacting with myeloid and lymphoid cell subsets, through direct cell-to-cell contact, secretion of soluble factors, EVs, etc. It has been shown that MSC action can be either immunostimulatory or immunosuppressive, depending on the context. In the case of B-ALL, the majority of reports suggest a general immunosuppressive effect of MSC that could promote leukemia immunoescape. However, whether MSCs, under specific conditions, could act as a promoter of an anti-leukemia action is still under debate.

In this regard, Entrena and colleagues [144] observed that MSCs isolated from B-ALL patients stratified as low- and moderate-risk, based on ongoing clinical protocols, were able to promote an anti-tumor NK cell response, including cytotoxicity. On the contrary, MSCs isolated from high-risk B-ALL patients, that are indeed characterized by a poor prognosis, suppressed the anti-tumor function of NK cells, suggesting a context-dependent action [144]. Alongside, it has been recently published that high-risk B-ALL patients show a reduced frequency of NK cells [145]. These NK cells, compared with their normal counterparts, exhibit an activated signature that is characterized by high CD56, but are less cytotoxic, and they positively correlate with poor clinical prognosis [144,145]. Concerning T cells, a recent paper from Zanetti and colleagues suggests that ALL-MSCs are able in vitro and in vivo, in a mouse model of severe colitis, to suppress T cell-mediated response similarly to HD-MSCs [146]. Interestingly, this immunosuppressive action is not exerted on CD19-CAR T cells [146].

Growing evidence highlights the presence within the B-ALL BM niche of many other immune effectors that are altered in terms of number and/or function. Indeed, independent studies reported the remodeling of the myeloid compartment in the PB and BM of B-ALL patients [60,147]. In this regard, myeloid-derived suppressor cells (MDSCs) were increased in B-ALL patients and the number of MDSCs belonging to the granulocytic subset correlated with therapy response [148,149]. In addition, the frequency of regulatory T cells was shown to be increased in B-ALL patients compared to controls [150,151] and to correlate with patients’ response to an immunotherapy strategy based on the administration of the anti-CD19 bispecific antibody, Blinatumomab [152]. Overall, a corrupted immune infiltrate seems to be a characteristic feature of the B-ALL BM. However, there is no clear data about a direct MSC-dependent immunomodulatory action that could be responsible for these alterations in the BM microenvironment.

## 3. Conclusions and Future Perspective

So far, research has been focused on finding new strategies to directly kill leukemic cells, either by finding new targets or by developing new immuno- and cellular-therapy approaches (e.g., monoclonal antibodies, CAR T-cell). The recent technological development that rendered whole genome and transcriptome analyses cost-affordable for specialized laboratories has opened the era of personalized medicine. As a consequence, mutation or fusion-specific drugs are under evaluation in combination with classical chemotherapy regimen in the attempt to improve the cure rates and avoiding chemotherapy dose intensification [153]. Besides these clinical advances, the role of the BM microenvironment in determining the response to therapy and the consequential patient outcome is becoming increasingly prominent. An increasing body of data has clearly proved that the BM microenvironment can provide chemoresistance to B-ALL cells. Furthermore, in the context of several solid and hematological cancers, immunotherapy-based studies are clearly highlighting that an immunosuppressive tumor microenvironment can be one of the most important factors determining the failure of either anti-tumor CAR-T cells [154] or monoclonal antibodies [152]. In this context, MSCs are acquiring ever-growing importance and the study of the mechanisms exploited by these cells to protect B-ALL blasts can be instrumental to find new ways to contrast leukemia and improve the effectiveness of leukemia-targeting strategies. As so far reviewed, the study of the bi-directional crosstalk between MSCs and leukemic cells has produced a number of possible druggable candidates to target the leukemic BM microenvironment to be coupled with anti-leukemia therapies to improve the prognosis of r/r patients, whose management represents an unmet medical need. Metabolic exchanges between MSCs and B-ALL cells, altered chemokine and cytokine axes, adhesive interactions and downstream activated molecular pathways are some of the targets with specific compounds already under experimentation (Table 1).

Lastly, the study of the leukemic BM niche has shed new light on the crucial impact of the surrounding microenvironment on leukemia eradication. Based on this evidence, it is clear how the study of other extramedullary niches, which are sites of leukemic metastasis, will be fundamental to ameliorate the response to therapy and ultimately the prognosis of relapsed patients. In this context, recent studies proved that the CNS microenvironment is able to establish, similarly to MSCs within the BM niche, adhesive interactions that promote leukemic blast chemoprotection [155]. Indeed, the study of the crosstalk between BM-MSCs and leukemic blasts in the BM is paradigmatic to identify potentially targetable pathways that could be involved in the maintenance and chemoprotection of the leukemic cell, also when residing in extramedullary sites.

## Figures and Tables

**Table 1:** Possible therapeutic approaches for targeting the B-cell acute lymphoblastic leukemia (B-ALL) bone marrow microenvironment.

Target	Compound	Mechanism of Action	Phase of Study/ Disease Approval	Clinical Trial Number ^1^
Asparagine	ASNase	Asparagine depletion	Approved by regulatory agencies for ALL treatment and non-Hodgkin’s lymphoma. In clinical trials for T-ALL, AML, MDS, etc.	NCT00501826, NCT00369317
Arginine	BCT-100	Arginase depletion	Clinical Trial for r/r ALL, r/r AML, hepatocellular carcinoma, melanoma and prostate adenocarcinoma	NCT03455140, NCT02089763, NCT02285101
TNTs	Vincristine	Disruption of actin cytoskeleton (TNTs disassembly)	Approved by regulatory agencies for B-ALL treatment and in clinical trials for advanced follicular lymphoma, non-Hodgkin’s lymphoma, Mantle Cell Lymphoma etc.	NCT03817853, NCT00911183, NCT05051891
CXCR4/CXCL12 axis	Plerixafor	CXCR4 inhibitor	Clinical Trial for r/r B-ALL, AML and MM.	NCT01319864, NCT02605460, NCT00903968
CXCR4/CXCL12 axis	BTK140	CXCR4 inhibitor	Clinical trial for T-ALL/lymphoma and r/r AML.	NCT02763384, NCT01838395
CCR4/CCL22 axis	Mogamulizumab [55]	CCL22/CCR4 inhibitor	Approved by regulatory agencies for r/r mycosis fungoides and Sézary syndrome. Clinical trials for peripheral and cutaneous T-cell lymphoma and adult T-cell leukemia/lymphoma and advanced/metastatic solid tumors.	NCT04745234, NCT00888927, NCT04848064, NCT01929486
PKC isoforms	HKPS	Chimeric peptide	Pre-clinical research.	N.A
ITGA4	Natalizumab	Monoclonal antibody	Approved by regulatory agencies for multiple sclerosis and Crohn’s disease. Clinical trial for osteosarcoma and multiple myeloma, and pre-clinical evaluation for B-ALL.	NCT03811886, NCT00675428
Proteasome inhibitor	Bortezomib	Interference with B-ALL/MSCs interaction	Approved by regulatory agencies for MMtreatment and in Clinical Trials for leukemias and diffuse large B cell lymphoma.	NCT03811886, NCT00675428
TGFBR1 inhibitor	Vactosertib	Inhibits the intracellular signaling of TGFβ	Clinical trial for colorectal cancer and blood cancers.	NCT05400122

^1^ Where more than one clinical trial is registered for the same agent, one of the most recent is reported for each cited pathology in the Table 1. Abbreviations: asparaginase (ASNase); T-cell acute lymphoblastic leukemia (T-ALL); acute myeloid leukemia (AML); myelodisplastic syndrome (MDS); relapsed/refractory (r/r); tunneling nanotubes (TNTs); multiple myeloma (MM); protein kinase C (PKC); ɑ4 integrin (ITGA4); mesenchymal stromal cells (MSCs); tranforming growth factor receptor 1 (TGFBR1); transforming growth factor β (TGFβ).
